# Malignant skin lesions in Oshogbo, Nigeria

**DOI:** 10.11604/pamj.2015.20.253.2441

**Published:** 2015-03-17

**Authors:** Ganiyu Oyediran Oseni, Peter Babatunde Olaitan, Akinwumi Oluwole Komolafe, Olaejirinde Olaniyi Olaofe, Hezekiah Adebola Morakinyo Akinyemi, Oreoluwa Adeola Suleiman

**Affiliations:** 1 Burns and Plastic surgery unit, Department of Surgery, Osogbo, Osun State, Nigeria; 2 Department of Histopathology, Ladoke Akintola Univeersity of Technology Teaching Hospital, Osogbo, Osun State, Nigeria

**Keywords:** Skin malignancies, cancers, carcinogens, dermatofibrosarcoma, squamous cell carcinoma, Basal cell carcinoma, malignant melanoma

## Abstract

**Introduction:**

The aim of this study is to retrospectively assess the prevalence of some of skin malignancies in our environment and to provide a data base for creating awareness for prevention and early detection of the diseases in order to reduce morbidity and mortality associated with these skin lesions in our environment.

**Methods:**

This is a retrospective study of all histologically diagnosed malignant skin lesions which presented at Ladoke Akintola University of Technology Teaching Hospital Osogbo Osun State between January 2006 and December 2010.

**Results:**

Ninety- eight patients presented with skin cancers out of which 60 (61.2%)were males and 38 (38.8%) were females. Malignant melanoma ranked highest followed by squamous cell carcinoma, dermatofibrosarcoma and basal cell carcinoma in that order. Malignant melanoma affects male more than female and it commonly affects lower limbs.

**Conclusion:**

Skin malignancies pose a burden to the economy of the country. Efforts should be directed toward prevention, early diagnosis and management in order to abolish or reduce morbidity, as well as mortality associated with late presentation of people in the developing countries.

## Introduction

Malignant skin lesions are the most common cancers in the white population [1] and happen to be among the leading cancers among the negroid population [1–7]. This is not surprising because skin happens to be the largest organ in the body and it is in contact directly or indirectly with a lot of carcinogens like ultra-violet radiation, chemical carcinogen and so on, in the environment. Most commonly encountered malignant skin lesions include squamous cell cancer (SCC), Basal cell cancer (BCC), malignant melanoma and dermatofibrosarcoma. Others include Karposis sarcoma, pleomorphic sarcoma, fibrous histiocytoma, malignant skin adnexae and a lot of others. BCC and SCC, also known as Non melanoma skin cancer (NMSC) have low mortality rate but morbidity is of great concern. In contrast, malignant melanoma has a very high mortality and morbidity rate and accounts for approximately 75% of all deaths from skin cancer [2]. Intensive UV light and radiation exposure, chronic irritation and unstable scars are some of the factors implicated in the development of most of these skin malignancies [7]. Histopathologic analysis remains the gold standard for diagnosis of these skin malignancies. The current study is therefore, necessary to review the different types of skin amlignancies that are commonly seen in our environment so as to plan preventive campaign and measures and educate the populace appropriately.

## Methods

This is a retrospective study of malignant skin lesions seen at Ladoke Akintola University of Technology Teaching Hospital Osogbo Osun State between July 2006 and June 2010. All patients with histologically confirmed skin malignancies managed during this period were included in this study. Relevant information was obtained from patient case notes, clinic record, operation notes, ward and histopathology registers. The data obtained included bio-data, location of the tumors and histological types. The results were presented as means, frequency tables, and figures as appropriate.

## Results

One thousand seven hundred and ninety patients were managed for various form of malignancies at Ladoke Akintola University of Technology Teaching Hospital Osogbo Osun State within the study period. Of this, ninety-eight patients were histologically diagnosed as skin malignancies (5.5%). This constitutes 60 (61.2%) males and 38 (38.8%) females with male: female ratio of 1.6:1. Malignant melanoma 37 (37.8%) and squamous cell carcinoma 32 (32.7%) constitute the most frequent skin tumours. Dermatofibrosarcoma 7 (7.1%) and basal cell carcinoma were found among 5 (5.1%) of the patients respectively. Other tumours observed include Karposis sarcoma in 3 (3.1%), pleomorphic sarcoma 3 (3.1%), malignant fibrous histiocytoma, 3 (3.1%), Cutaneous Non-Hodgkin's Lymphoma, 2 (2.0%). Others are as shown in Table 1. Melanoma was observed in 22 (59.5%) of the cases among males and 15 (40.5%) females (Male: Female of 1.5:1). A closer look at the malignant melanomas revealed that the right side of the body was involved in 21 (56.8) and the left, 14 (37.8%). The distribution was as shown below with the right foot, 15 (40.5%) and the left foot, 11 (30.6%), the right leg 5(13.9%), the left leg 2 (5.6%), the head and neck 1, (2.8%), trunk, 1 (2.8%), and the anus, 1 (2.8%) (Table 2), (Table 3).

**Table 1:** Histological types of malignant skin lesions

Histology	Male	Female	Total
Malignant melanoma	22	15	37
Squamous cell carcinoma	19	13	32
Basal cell carcinoma	3	2	5
Dermatofibrosarcoma protuberance	3	1	4
Karposi's sarcoma	2	1	3
Pleomorphic sarcoma	1	2	3
Malignant fibrous histiocytoma	2	1	3
Cutaneous Non Hodgkin Lymphoma	3	-	3
Malignant skin adnexa tumours	3	-	3
Epithelioid sarcoma	-	2	2
Mucinous carcinoma	1	-	1
Myxoid liposarcoma	1	-	1
Adenocarcinoma	-	1	1
TOTAL	60	38	98

**Table 2:** Parts of the body affected by different major skin cancers

Part of the body affected	Squamous cell carcinoma	Basal cell carcinoma	Malignant melanoma	Dermato-fibrosarcoma
Head and neck	8	4	-	-
Upper limb	6	-	-	-
Leg and thigh	10	-	4	2
Trunk	2	1	4	5
Genitalia	3	-	-	-
Foot	3	-	29	-
**TOTAL**	**32**	**5**	**37**	**7**

**Table 3:** Other rare skin cancers and body distribution

Tumour Types	Part of the body affected			
	Upper Limbs	Lower Limbs	Trunk	Foot
Karposis sarcoma	-	2	-	1
Pleomorphic sarcoma	-	2	-	1
Malignant fibrous histiocytoma	-	-	3	-
Malignant skin adnexa	-	-	3	-
Myxoid liposarcoma	-	-	1	-
Cutaneous Non- Hodgking's Lymphoma	-	-	3	-
Epitheloid sarcoma	2	-	-	
Mucoid carcinoma	-	-	-	-
Malignant fibrous histiocytoma	-	-	3	-

## Discussion

Recent publications have shown an increase in the incidence of skin malignancies all over the World [5–8]. This may not be unconnected to depletion in ozone layer [9] and lack of adequate knowledge on the prevention and treatment of skin malignancies in the developing countries like Nigeria [2, 4, 8, 9]. The prevalence of skin malignancies in this study is 5.5%. This is comparable with prevalence of 6.18% in jos but lower than 10% in Calabar, 12.3% in Zaria and 12.7% in Kano. The higher prevalence in Zaria and Kano may be explained by high environmental temperature and low humidity in those two cities [10–12]. Malignant melanoma 37 (37.8%) is the most common skin malignancy in this report, followed by Squamous cell cancers 32 (32.7%). This is in contrast to the reports from United State and other Western world which favor basal cell cancer as the most common skin malignancies [10–14]. The reason for high preponderance of squamous cell carcinoma in this report is not known. However, chronic wounds and poorly managed scars and burns that are common in this environment may be responsible for the high prevalence of squamous cell carcinoma [15], while the malignant melanoma which is quite common on the foot has not had any scientific explanation for its preponderance at this site among the blacks. Although, recurrent trauma to this part of the body, which is also common among the people of this part of the world, has been advocated as one of the reasons for high preponderance of melanoma on the sole of the foot [10, 14–18]. However, further scientifically provable works will need to be done to provide reasons for this. While considering skin cancers generally in this study, men were affected more than women. This is similar to finding by many authors in Africa [10, 16, 19–21]. Outdoor activities of the men can partly explain this preponderance but there may be other reasons for this. Dermatofibrosarcoma protuberance, which is a rare skin tumor is the third most common skin malignancy in this report. Many reports have shown a higher incidence of dermatofibrosarcoma in blacks compare to their white counterparts [22]. Dermatofibrosarcoma protuberance affected more male, 4(57.1%) than female, 3 (42.9%) as reported by many authors [11, 12, 23, 24]. Trunk is affected in all the patients in this report. Basal cell carcinoma represents 5 (5.1%) of skin malignancies. This is in contrast to 70-80% in white but comparable with what has been reported in Nigeria and other African countries [2, 3, 6, 10, 25]. The low incidence of basal cell carcinoma may be as a result of protective function of melanocytes present in black race. Kaposis sarcoma was rare in Africa before the advent of HIV. However, the incidence of Kaposis sarcoma has been found to parallel HIV infections [10, 14, 26]. The incidence of Kaposis sarcoma was found to be 3 (3.1%) in this report and all of them were found to be HIV positive. Skin malignancies pose a big burden to the patient, patients’ relatives, the Health care delivery system and economy of the country. Late presentation is a problem of patients with malignant lesions generally in our country [27] most especially when the lesion is usually not painful at the beginning.

## Conclusion

Efforts on prevention and routine screening toward early detection of the disease would make a great impact in reducing the prevalence of the disease in the society as well as reducing morbidity and mortality associated with late presentation of the patients with these diseases in some of the centers in the developing Countries. Increasing the knowledge of the populace about the need to present to the hospital with every lesion on the skin especially when these continue to grow in spite of whatever treatment has been offered is also very important.
